# Value-directed learning: Schematic reward structure facilitates learning

**DOI:** 10.3758/s13421-023-01406-6

**Published:** 2023-03-09

**Authors:** Katie M. Silaj, Karina Agadzhanyan, Alan D. Castel

**Affiliations:** Department of Psychology, University of California, Los Angeles, CA 90095 USA

**Keywords:** Value-directed remembering, Schematic support, Learning, Metacognition

## Abstract

When learning, it is often necessary to identify important themes to organize key concepts into categories. In value-directed remembering tasks, words are paired with point values to communicate item importance, and participants prioritize high-value words over low-value words, demonstrating selective memory. In the present study, we paired values with words based on category membership to examine whether being selective in this task would lead to a transfer of learning of the “schematic reward structure” of the lists with task experience. Participants studied lists of words paired with numeric values corresponding to the categories the words belonged to and were asked to assign a value to novel exemplars from the studied categories on a final test. In Experiment 1, instructions about the schematic structure of the lists were manipulated between participants to either explicitly inform participants about the list categories or to offer more general instructions about item importance. The presence of a visible value cue during encoding was also manipulated between participants such that participants either studied the words paired with visible value cues or studied them alone. Results revealed a benefit of both explicit schema instructions and visible value cues for learning, and this persisted even after a short delay. In Experiment 2, participants had fewer study trials and received no instructions about the schematic structure of the lists. Results showed that participants could learn the schematic reward structure with fewer study trials, and value cues enhanced adaptation to new themes with task experience.

## Introduction

As we are often exposed to large amounts of information, people must be selective in what they choose to remember, often at the cost of other information. Research has demonstrated that participants can selectively remember important information when paired with a numeric value, a phenomenon known as *value-directed remembering* (VDR; Castel et al., 2002; see Knowlton & Castel, 2022, for a review). Value has a direct influence on the selective encoding of more important information over less important information (Castel et al., 2007), and the ability to succeed on a typical VDR task is related to the strategic control of memory processes (Hennessee et al., 2019).

Meaningful learning occurs when a person can interpret new information, incorporate it with prior knowledge, and apply it to novel problems (Lujan & DiCarlo, 2006). One way to make novel information more meaningful is to incorporate existing schemas, or general knowledge structures consisting of bits of information obtained through experience that guide a person’s understanding of a particular concept. Schemas support learning through their influence on retrieval processes and memory reconstruction, and their role in attentional and encoding processes (Bartlett, 1932; Graessner & Nakamura, 1982; Webb & Dennis, 2019). When schemas are used during learning, they can provide the background knowledge necessary to make inferences and formulate predictions in novel situations (Norman & Bobrow, 1976). For example, prior knowledge (a form of “schematic support”; Craik & Bosman, 1992) can influence memory performance when learning the prices of common grocery items (Castel, 2005). Specifically, when grocery items were associated with realistic prices (market value), older adults showed similar memory performance for the studied prices as younger adults, but when studying items associated with unrealistic prices (overpriced), younger adults outperformed older adults (Castel, 2005). Furthermore, both age groups were able to identify the general category of the prices of each item and use prior knowledge to predict the new item values. Other work has shown that younger adults also benefit from schematic support when learning (Kuhns & Touron, 2020), and schemas can make learning easier even without employing strategic control processes (Whatley & Castel, 2022).

When accompanying schemas, value can communicate meaning beyond item importance. In VDR tasks, words are paired with values, and participants are instructed to prioritize high-value over low-value words (Castel et al., 2002). Typically, the words used in VDR tasks are unrelated to each other; however, if participants are presented with a series of words paired with point values based on category membership, they may notice that values repeated in the word list are connected to similarities between words sharing the same value. This process may lead to a realization of the existence of categories within the word lists, as categories are used in classifying new objects into known groups of distinct items that share similar properties (Markman & Ross, 2003). For example, when encountering words, such as “parrot,” “owl,” and “raven” paired with a high numeric value indicating their importance, one might notice similarities between them, leading to a grouping of those words into a category of “birds,” which share similar properties, like the presence of feathers. One might also notice differences between words from the “bird” category and other words, like “carp,” “tuna,” and “shark” paired with a lower numeric value, which belong to the category “fish,” sharing similar properties, like the presence of fins. Therefore, by allocating attention towards the higher value words, one learns not only that high-value words are important to remember, but also learns which words are paired with high values.

Categories can also be used to make predictions about new items using previous knowledge about the category to which each word belongs (Anderson, 1990) and experimental research has shown that people can learn categories without prior knowledge of category labels (Fried & Holyoak, 1984). Pairing numerical values with categories creates a *schematic reward structure* in which participants may learn how values are meaningfully paired with categories through being guided by the points they earn upon recall, extending the VDR paradigm to “value-directed learning (VDL).” Thus, if the next word “trout” is presented alone without a value, the participant may use their knowledge about this schematic reward structure combined with their prior knowledge about fish properties to predict the word to be associated with a low value, demonstrating a *transfer of learning* (e.g., Perkins & Salomon, 2012; Salomon & Perkins, 1989) of the schematic reward structure of the word lists. Such evidence of transfer of learning would demonstrate the combined effect of numerical value cues and schematic support on learning and memory. Specifically, because numerical values are often used in rewards (e.g., course grades, bonuses, scores in a baseball game), and prior research has demonstrated that rewards can be used to selectively guide attention (Chelazzi et al., 2013), these reward-dependent effects are strategic. Rewards can be used to allocate attention to objects, features, and locations that have been accompanied by rewards. This process requires active metacognitive processes, such as metacognitive monitoring of memory processes and control of future behavior based on this monitoring (Dunlosky & Metcalfe, 2009). If rewards are assigned to items based on category membership, metacognition should play a role in decision-making about which new items will be valuable to remember based on experience studying the schematic reward structure of previously encountered items.

While metacognitive monitoring refers to self-assessments of learning such as judging whether you will remember a given word on a future test, metacognitive control refers to the self-regulation of learning based on information gained from monitoring, such as choosing which information to study in preparation for an exam (Dunlosky & Mueller, 2016; Nelson & Narens, 1990; Son & Metcalfe, 2000; Thiede & Dunlosky, 1999). For example, after studying each word within a list, people may predict whether they will recall those words on a later test by making item-level judgments of learning (JOLs; see Rhodes, 2016, for a review) and after receiving feedback about their performance on a list, people may use the feedback to adjust their studying and boost their performance on the later lists. Thus, learning through engaging in metacognitive monitoring can aid in identifying the association between stimuli and the reward associated with them. The use of rewards to facilitate selective attention in subsequent tasks requires active monitoring of performance, but metacognitive monitoring during study may also contribute to learning. Previous work has demonstrated that making global JOLs before the learning session can result in a higher transfer of learning compared to making local item-level judgments after studying each word (Lee & Ha, 2019).

Although engaging in metacognitive judgments is potentially important for recognizing patterns within trials and applying them in novel situations, differences in fluid intelligence may also play a role. Raven’s Progressive Matrices (RPM; Hall, 1957) is a test of fluid intelligence, which measures the ability to reason and succeed in tests that require adaptation to novel situations (Cattell, 1963). Prior work has shown that higher fluid intelligence scores attained through the RPM test are related to higher selectivity in a typical VDR task when the study time for the word lists was fixed (Murphy et al., 2021). We collected measures of fluid intelligence in Experiment 1 to explore whether extracting a schematic reward structure from a series of word lists relates to abilities such as problem solving, abstract thinking, and reasoning. Here we aimed to investigate the following research questions:

1. Recall performance: In a value-directed remembering experiment using value cues associated with categories (as opposed to being randomly paired with individual items), does metacognitive monitoring and control impact recall performance with task experience? Does fluid intelligence relate to the proportion of high-value words recalled with task experience?
2. Transfer of learning: On a value-directed learning task where participants are asked to predict the values of items based on their experience studying related items, is fluid intelligence related to word-value pairing accuracy? Does being given specific instructions of the categories present in the word lists prior to beginning the experiment lead to higher accuracy? Do visible value cues paired with words on the studied lists result in higher accuracy on the transfer of learning task? Does the effect of value cues on transfer performance depend on the type of schema instructions provided?

## The Current Study

In the current study, we examined the effects of value cues and schematic support on learning in a VDL task. Specifically, in Experiment 1, participants studied word lists in which each word belonged to a specific category. Within the lists, words from a given category were associated with a point value indicating their importance. Half of the participants received specific instructions about the schematic reward structure of the word lists before beginning the task, while the other half were not made explicitly aware of the categories. Furthermore, half of the participants studied words paired with visible values during encoding while the other half studied words alone. We chose to scaffold support provided to participants to model how learning in classroom contexts and other realistic environments is often facilitated by different types of motivation. For example, some learners may benefit from the extrinsic reward of points earned upon recalling high-value words. This may lead them to notice similarities between words sharing the same value. Other learners may benefit from being reminded of their prior knowledge of a topic. For example, being told that to-be-studied-content will contain items from categories the learner has prior knowledge of may make them more aware of the categories as they study.

After encoding each of the lists, participants provided global JOLs and completed a free recall test. After following this procedure for five lists, participants were then presented with novel words belonging to the studied animal categories and were asked to assign a value to each item based on the prior lists (immediately in Experiment 1a and after a short delay in Experiment 1b), measuring their transfer of learning. In Experiment 2, we did not provide explicit instructions about the schematic reward structures of the word lists but did manipulate the presence of value between participants. Furthermore, participants were presented with a new theme with each trial, requiring them to learn the schematic reward structures with fewer trials and adapt to new categories throughout the task.

## Experiment 1a

In Experiment 1a, the type of instruction and the presence of value was manipulated between participants. Participants were either given general or specific instructions about the schematic nature of the lists and either studied the words paired with visible values or alone. After studying and recalling five lists of animal words divided into three categories where each category was associated with a low, medium, or high value, participants engaged in a final transfer task. Participants also completed a test of fluid intelligence after the transfer task to examine whether fluid intelligence is related to transfer of learning.

- Hypothesis 1 (H1): When given specific schema instructions, we expected participants to demonstrate a higher transfer of learning than those who were given general instructions as participants may benefit from an explicit cue to activate their prior knowledge of the categories within the word lists (Castel, 2005).
- H2: Similarly, we expected participants who studied words paired with visible values to demonstrate a higher transfer of learning than those who studied the words alone. Allocating attention towards words paired with high values may motivate participants to notice similarities between words paired with the same value, activating their prior knowledge of the categories.
- H3: Participants receiving general instructions with no value cues were included as a control condition, thus we expected them to perform at chance in both the VDR task and VDL task with no difference between the other conditions in recall performance. Additionally, we expected all other conditions to perform significantly better than this control condition on the VDL task. Finally, we expected participants receiving both specific instructions and visible value cues to demonstrate a performance advantage on the transfer task compared to all other conditions. Being given information about the categories present in the word lists prior to beginning the task eliminates the need for the participant to discover these categories as they study the word lists. Furthermore, being reminded of what points are associated with each category throughout the task by studying the words paired with visible value cues frees up space in working memory so the participant’s attention will not be divided between discovering the categories present in the word lists and binding the categories with their associated point values.
- H4: Extensive prior work using the VDR paradigm has shown that value cues influence selectivity in recall (Castel et al., 2002; Knowlton & Castel, 2022; Middlebrooks & Castel, 2018). Therefore, though this application of the VDR paradigm is novel (binding categories of words with certain values), we expected participants receiving value cues to recall a higher proportion of high-value words compared to participants who did not receive value cues during encoding.
- H5: We expected participants who made higher global JOLs to recall more words with task experience as metacognitive monitoring can lead to metacognitive control when feedback is provided on performance (Lee & Ha, 2019).
- H6: Murphy et al. (2021) found that higher fluid intelligence was related to recalling more high-value words in a VDR task. Here we explore the influence of fluid intelligence on our transfer task where participants are expected to predict the values that are associated with each word based on their experience with similar items. This work is exploratory as there is no prior work investigating this specific association; however, we expect higher fluid intelligence to be associated with higher transfer scores.

### Method

#### Participants

Participants were 120 undergraduate students (age: 18–38 years, *M* = 20.03, *SD* = 2.60; gender identity: 90 women, 27 men, one nonbinary, two prefer not to say) recruited from the University of California Los Angeles (UCLA) Human Subjects Pool who were tested online and received course credit for their participation.^1^ Because our task involves categorizing English nouns we asked participants whether they were fluent in English and how old they were when they began learning English. On average, participants began learning English at 1.83 years (*SD* = 2.87). The sample size was selected based on prior exploratory research and the expectation of detecting a medium effect size (Knowlton & Castel, 2022; Schwartz et al., 2023). A sensitivity analysis based on the observed sample was conducted using G*Power (Faul et al., 2009). For a multiple linear regression (MLR) with 6 predictors, assuming alpha = .05, power = .80, the smallest effect the design could reliably detect is η2 = .11.

#### Materials

Stimuli used in the experiment consisted of 90 English animal names (see Appendix 1 for word lists used in Experiment 1). When schemas already exist, memory consolidation can happen more quickly (Tse et al., 2007), so using well-known categories may be a more effective way to assess whether a schematic reward structure can be learned and applied in a relatively short laboratory task than using nonwords and novel categories. Because our sample consisted of college students who were fluent in English, we expected them to be familiar with English animal words and be able to identify common categories of animals such as mammals, birds, and fish. These words were submitted to the English Lexicon Project (ELP; Balota et al., 2007) database to generate measures of length (*M* = 6.02 letters per word, *SD* = 1.75), frequency in the Hyperspace Analogue to Language corpus (HAL; Lund & Burgess, 1996; *M* = 6.86 occurrences per million, *SD* = 1.64), and concreteness (*M* = 4.76, *SD* = 0.27). Each animal name belonged to one of three categories: mammals, birds, or fish. There were five animals from each category per list, and each word was associated with a value of either 1, 3, or 5, signifying the importance of the word (1 = low importance, 3 = medium importance, 5 = high importance) based on animal group. Category-value pairings were counterbalanced.

#### Procedure

A 2 (Value: No Value Cue, Value Cue) × 2 (Schema: General Instructions, Specific Instructions) design was used, with all factors manipulated between participants. All participants were told that they would study six lists of words that they would later be asked to recall and that each word was associated with a value of either 1, 3, or 5. They were also told that their goal was to maximize their scores which would be based on the sum of the points associated with the words they recalled and to try to remember as many words as they could. Additional instructions were provided to participants based on their randomly assigned conditions (see Table 1): No Support, Value Support, Schema Support, and Dual Support. Value cues during encoding were either present or absent. If value cues were present, participants were instructed that each word would be paired with a value of 1, 3, or 5 and that words paired with 5 were most important. If value cues were absent, participants were told they would not be able to see the values paired with each word but were aware that some words were worth more points than others. Instructions about the schematic reward structure were either specific or general. Participants receiving specific schema instructions were informed that each word belonged to one of three categories: animals, birds, or fish. They were also told that how many points each word was worth depended on its category and were given the category-value pairings (e.g., “mammals are worth 5 points”). Participants receiving general schema instructions were not informed of the animal categories.

**Table 1:**
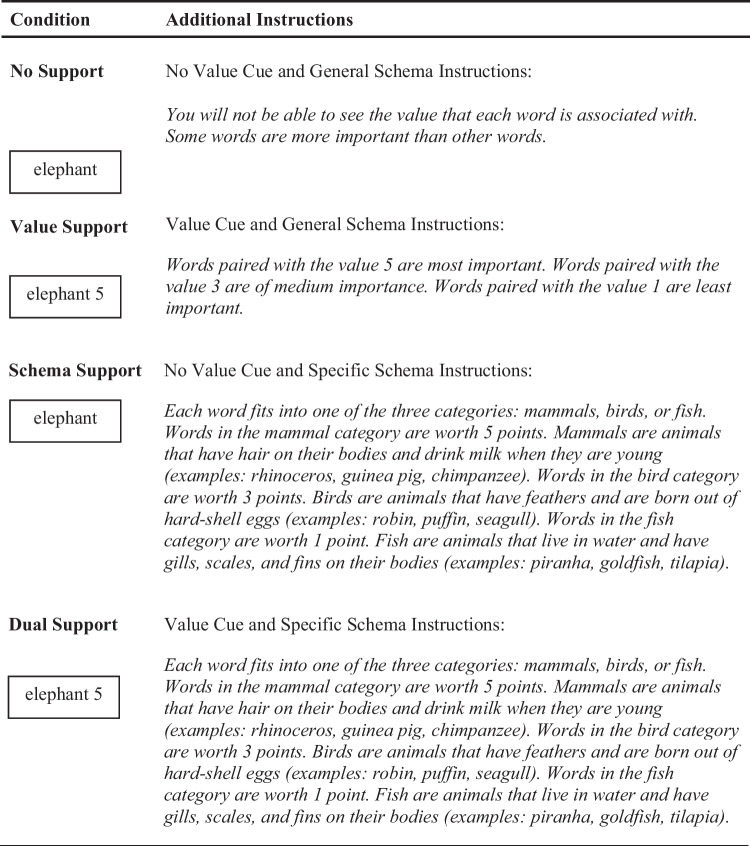
Instructions for the study phase of the value-directed learning task for each condition

**Fig. 1:**
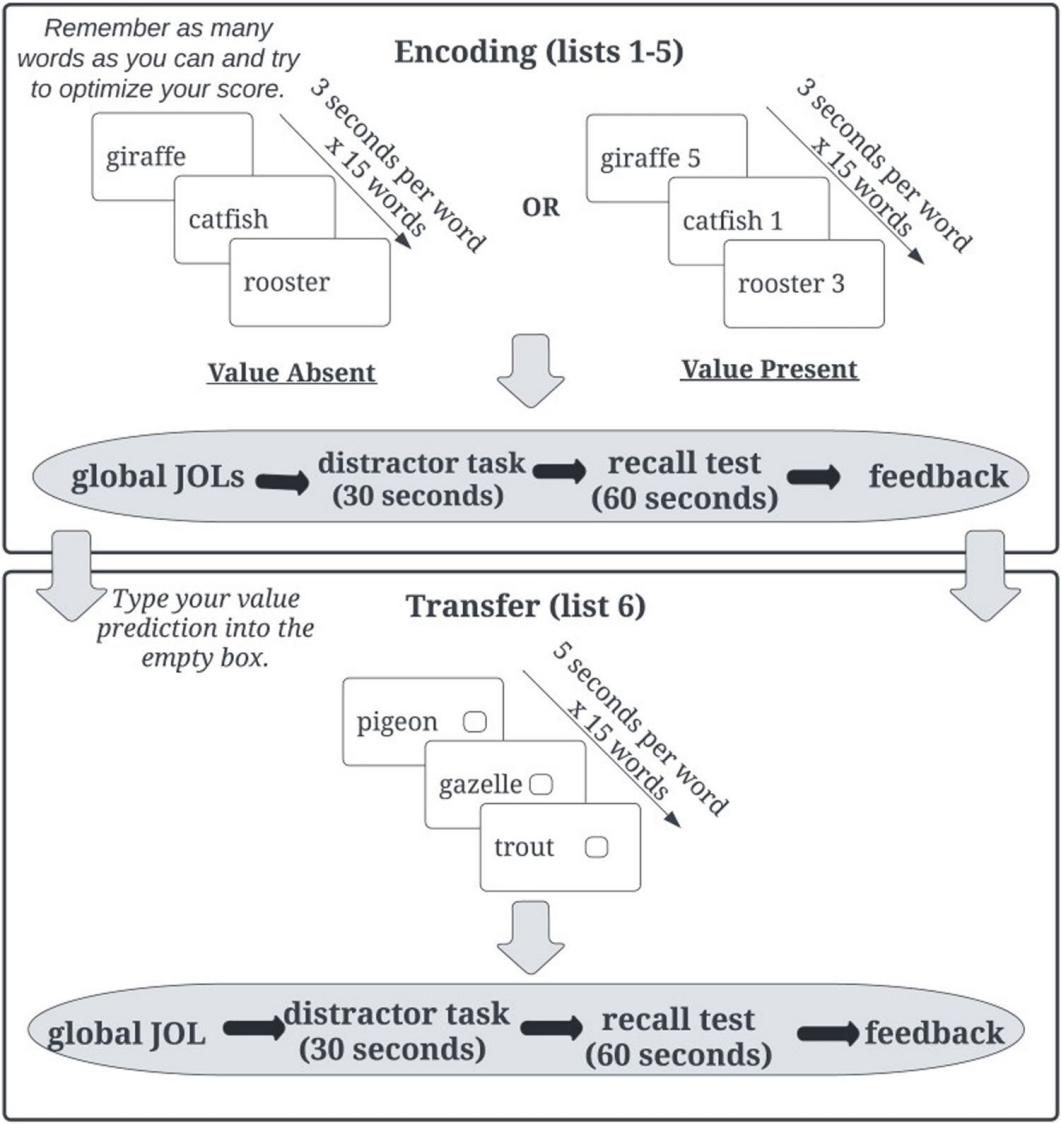
Procedure for the encoding and transfer phases of the value-directed learning task in Experiments 1a and 1b

The procedure for Experiment 1a is illustrated in Fig. 1. After studying all 15 words within a list, participants were asked to make a global JOL: *“What percentage of words do you think you will be able to recall in a few minutes?”* Immediately following the JOL, participants completed a 30-s distractor task where they had to reorder randomly generated sets of three numbers from largest to smallest (Unsworth, 2007). Following the distractor task, participants had 1 min to complete a free recall test by typing as many words as they could remember from the previously studied list. Participants were then presented with their score out of a possible 45 points (five 5-point, 3-point, and 1-point words per list). We used a real-time textual similarity algorithm to account for typographical errors in participants’ responses on the free recall tests for all experiments presented in this paper. Responses with at least 75% similarity to the studied word were counted as correct (Garcia & Kornell, 2014). Participants followed the same procedure for a total of five lists.

After List 5, the encoding phase ended and participants received additional instructions for the transfer task: “In this final list, you will see a series of words, each paired with an empty box. Your goal is to predict which value belongs with each word based on the five previous lists you studied. You will have 5 seconds to type your value prediction into the empty box. You should assign each word a value of either 1, 3, or 5.” Participants had 5 s to type their prediction into the box next to each new exemplar to demonstrate transfer of learning. If participants failed to type a prediction in the box within 5 s, the trial was scored as incorrect and they moved on to the next item (on average, participants failed to type a prediction on 3.56% of trials in Experiment 1a, 5.39% of trials in Experiment 1b, and 4.89% of trials in Experiment 2). They then made a global JOL, completed the distractor task, free recall test, and lastly were presented with their recall score. Participants were never told how many values they correctly paired on the final list. After the transfer task, to measure their fluid intelligence, participants completed the RPM test (e.g., Jarosz et al., 2019; Staff et al., 2014) consisting of 12 patterns of varying difficulty, each of which had a piece missing. Participants were instructed to select the correct missing piece from eight multiple-choice options, and the timing was self-paced such that participants could spend as much time on each item as they liked.

### Results

We collected several measurements across Experiment 1a including global JOLs to measure metacognitive monitoring after studying each list. On the final list (i.e., the transfer test), participants were presented with novel animal exemplars falling into one of the same three categories present on the five studied lists: mammals, birds, and fish. For each presented item, participants were asked to type a value of either 1, 3, or 5 into the box next to the word to demonstrate a transfer of learning of the schematic reward structure of the lists. Scores on this task used in the following analyses are presented as the proportion of correct word-value pairings as a function of associated value (out of five trials per value). Finally, we also included fluid intelligence scores in some of the analyses which were calculated as the proportion correct (out of 12 trials) on the RPM task.

#### Recall

**Fig. 2:**
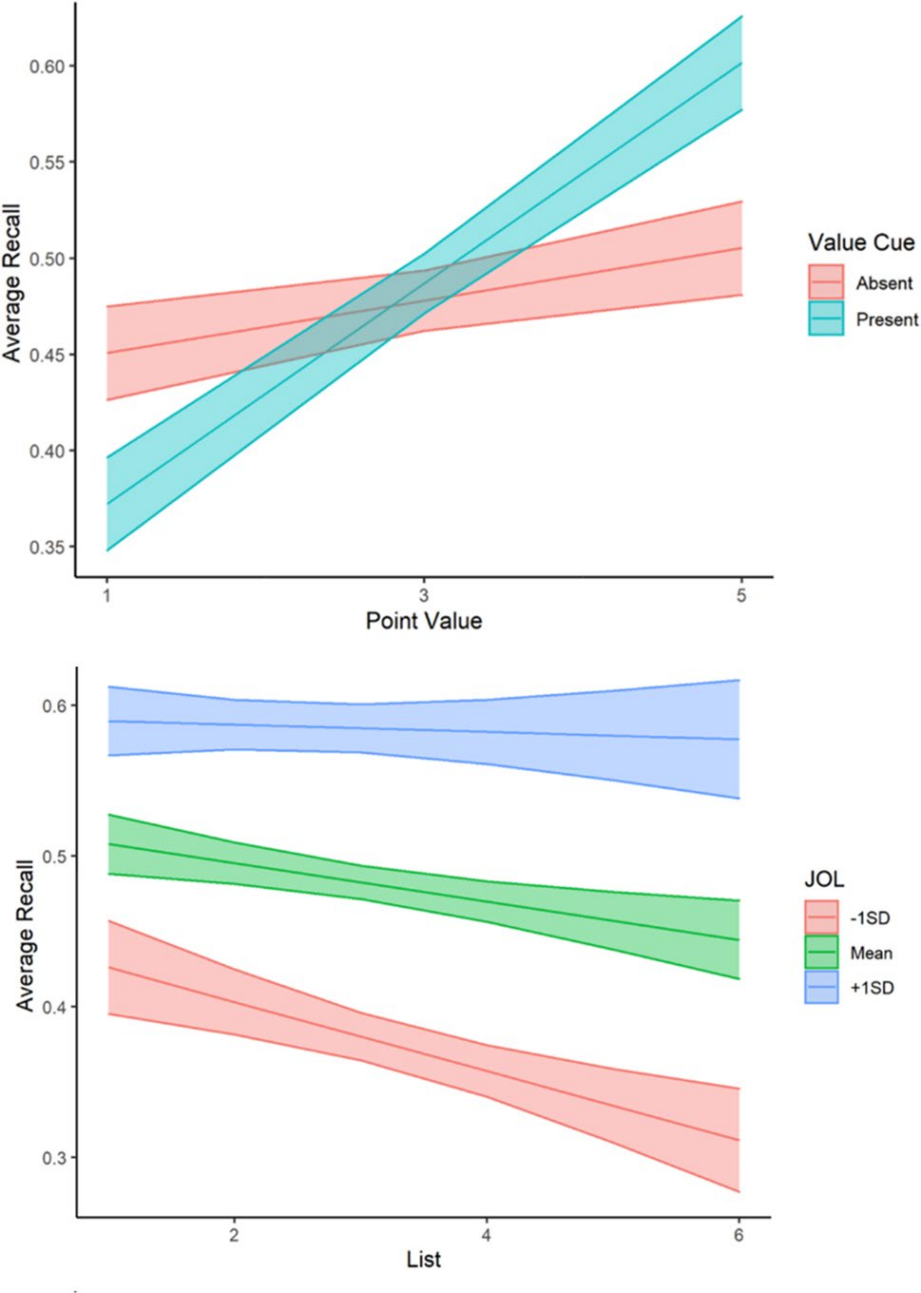
Recall performance in Experiment 1a. **Top graph:** Average recall as a function of point value and value cue condition. **Bottom graph:** Average recall as a function of list and average global judgment of learning. Confidence bands represent 95% confidence intervals for the predicted values of the mean

First, we sought to examine recall performance as a function of value cue, schema instructions, global JOL, list, and point value. We fit a MLR to model recall scores with value cue condition (no value cue = 0, value cue = 1), schema instruction condition (general instructions = 0, specific instructions = 1), point value, list, and global JOL. We also included interaction terms to examine both how value cue impacts the relationship between point value and recall and how metacognition impacts performance across lists. The model’s explanatory power (*R*^*2*^) was .23. The model’s intercept was at .34, *t*(1792) = 10.10, *p* < .001. The effect of schema instructions, *b* = .002, *t*(1792) = .19, *p* = .85, was non-significant, suggesting that those receiving specific instructions performed similarly to those receiving general instructions. All other predictors were significant: The effect of value cue was significant and negative, *b* = -.12, *t*(1792) = -5.30, *p* < .001, the effect of point value was significant and positive, *b* = .01, *t*(1792) = 2.87, *p* = .004, and the interaction between value cue and point value was significant and positive, *b* = .04, *t*(1792) = 6.49, *p* < .001. Therefore, while those receiving value cues during encoding recalled significantly fewer words on average, a one-point increase in point value resulted in a .01 increase in recall score and this effect was dependent on whether value cue was present during encoding (see Fig. 2). A simple slopes analysis revealed the effect of point value on recall was dependent on value cue condition such that those receiving value cues at encoding showed an increase of .06 in words recalled on average with each increase in point value, *b* = .06, *t*(1792) = -12.05, *p* < .001, and those studying the words alone still showed an increase in recall for higher-value words, but with a smaller slope, *b* = .01, *t*(1792) = 2.87, *p* = .004. Furthermore, the effect of average JOL was significant and positive, *b* = 0.34, *t*(1792) = 5.53, *p* < .001, the effect of list was significant and negative, *b* = -.03, *t*(1792) = -3.70, *p* < .001, and the interaction between JOL and list was significant and positive, *b* = .05, *t*(1792) = 2.62, *p* = .01. These findings suggest that holding all other predictors constant, on average, a one-unit increase in average JOL on the studied lists predicted a .34 unit increase in recall performance, and recall performance decreased by .03 units with each additional list. A simple slopes analysis revealed the effect of list on recall was dependent on JOL such that those with average JOLs at the mean (*M* = .39, *SD* = .18), *b* = -.01, *t*(1792) = -3.14, *p* = .002, and 1 standard deviation below the mean, *b* = -.02, *t*(1792) = -3.93, *p* < .001, recalled fewer words with each additional list, while those with average JOLs 1 standard deviation above the mean recalled a similar number of words across lists, *b* = -.002, *t*(1792) = -.45, *p* = .65 (see Fig. 2).

#### Transfer of Learning

**Fig. 3:**
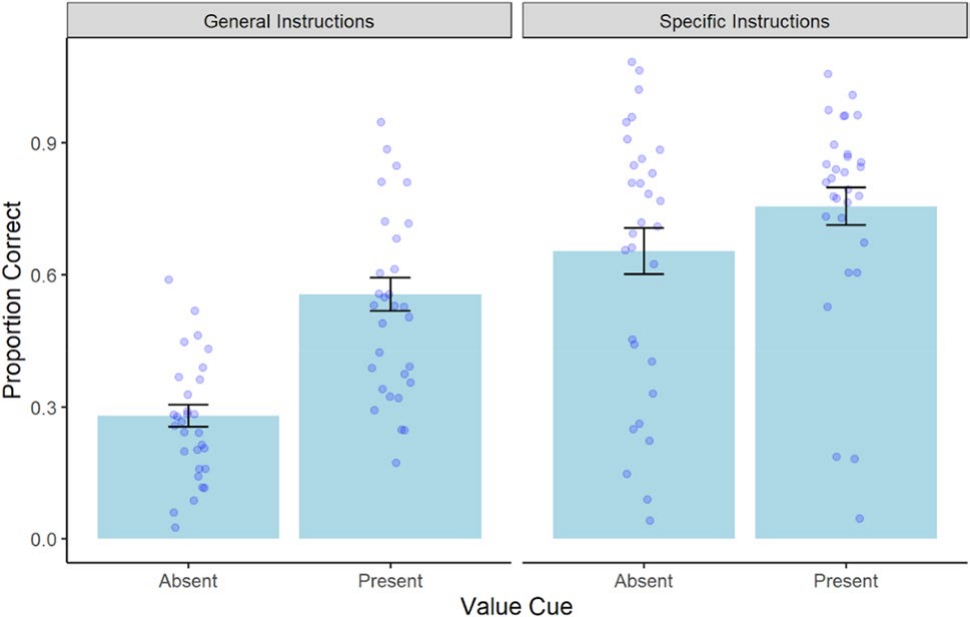
Average transfer scores as a function of value cue and schema instructions in Experiment 1a. Error bars represent the standard error of the mean

Next, we sought to examine transfer performance as a function of value cues, schema instructions, point value, and fluid intelligence. We fit a MLR to predict transfer of learning scores with value cues, schema instructions, point value, and fluid intelligence. We added interaction terms between schema instructions and value cues to evaluate whether the effect of value cues on transfer performance was dependent on the type of schema instructions participants received before beginning the task. We also added an interaction term between value cues and point value to test whether the effect of value cue on transfer performance was dependent on whether value cues were present during encoding. The model’s explanatory power (*R*^*2*^) was .33. The model’s intercept was at 0.29, *t*(323) = 6.05, *p* < .001. The effect of point value, *b* = -.01, *t*(323) = -.41, *p* = .69, fluid intelligence, *b* = .002, *t*(323) = 1.24, *p* = .22, and the interaction between value cue and point value, *b* = .004, *t*(323) = .23, *p* = .82, were not significant. Therefore, transfer performance was not significantly influenced by fluid intelligence, how many points each word was worth upon recall, and the effect of point value did not depend on the presence of value cues during encoding. All other predictors were significant: value cues, *b* = .27, *t*(323) = 3.76, *p* < .001, schema instructions, *b* = .43, *t*(323) = 10.19, *p* < .001, and the interaction between value cues and schema instructions, *b* = -.25, *t*(323) = -4.08, *p* < .001. On average, participants receiving value cues during encoding performed significantly better on the transfer task than those who studied the words alone. Similarly, participants receiving specific schema instructions had significantly higher transfer scores than those receiving general instructions. Furthermore, the effect of value cues on transfer performance depended on the type of schema instructions that were provided at the beginning of the experiment. Specifically, a simple slopes analysis revealed that when the schema instructions were specific, there was no additional effect of value cues on transfer performance, *b* = .03, *t*(1792) = .67, *p* = .50. However, when schema instructions were general, the presence of value cues during encoding resulted in significantly higher transfer performance compared to studying the words alone, *b* = .28, *t*(1792) = 6.45, *p* < .001 (see Fig. 3).

Because there were three possible point values participants were instructed to use as predictions of items on the transfer task, performing at chance on this task would be .33, or five items correctly paired with the appropriate point values. We conducted within-condition one-sample *t*-tests to examine whether each group performed better than chance and found that all groups receiving some form of support performed significantly better than chance on this task: Value Support (*M* = .56, *SD* = .28), *t*(89) = 7.42, *p* < .001, *d* = .78, Schema Support (*M* = .65, *SD* = .34), *t*(89) = 8.96, *p* < .001, *d* = .94, and Dual Support (*M* = .76, *SD* = .29), *t*(89) = 13.65, *p* < .001, *d* = 1.44. However, the No Support group who studied the words alone with general instructions performed below chance (*M* = .28, *SD* = .22), *t*(89) = -2.28, *p* = .03, *d* = -.24.

### Discussion

In Experiment 1a, we aimed to extend the VDR paradigm to category learning to investigate whether participants could learn the assignment of values to words based on category membership and could transfer their learning of the schematic reward structure of the lists on a final transfer task. We scaffolded instructions about the schematic nature of the word lists to either explicitly inform participants about the existence of categories and values within the lists or to provide general instructions about how some words were more important to remember than others. Results revealed that on average, participants who studied the words paired with visible value cues performed better on the transfer task than those who studied the words alone, confirming H2 that value cues would direct attention to the schematic structure of the word lists. However, those receiving value support recalled fewer words overall, but more high-value words compared to those studying the words alone. Thus, while value cues resulted in better value-based learning (H4), it is unclear specifically through what mechanisms these cues facilitated performance on the transfer task. Additionally, specific schema instructions at the beginning of the task supported performance on the transfer task compared to having general instructions (H1). Furthermore, it seems that the effect of value cues on transfer performance depended on the type of schema instructions participants received at the beginning of the task (H3). Those receiving both value cues and specific schema instructions performed significantly better than those receiving value cues and general schema instructions, but similar to those studying the words alone with specific schema instructions.

We also examined whether measures of metacognition during encoding influenced recall performance and found that overall, those with higher average JOLs also recalled more words. Furthermore, though recall decreased with each list, this was only the case for participants with low to average JOLs whereas those with higher JOLs maintained similar recall scores across lists. This result was not in line with H5 that higher JOLs would lead to higher recall with task experience, though metacognitive processes do seem to play a role in maintaining recall performance across lists. Finally, we were interested in how individual differences in fluid intelligence may relate to learning in our VDL task. Contrary to our prediction in H6, results showed that on average, fluid intelligence did not significantly impact transfer of learning. Thus, surprisingly, differences in the ability to think abstractly and solve problems in novel situations as measured by RPM was not related to the ability to succeed in learning the schematic reward structure and applying it in novel settings. Based on our findings, in Experiment 1b, we moved the RPM test between the encoding and transfer phases of our task to act as a distractor task as opposed to using it to measure fluid intelligence.

## Experiment 1b

Experiment 1b used the same materials and procedure as Experiment 1a except for two main changes: (1) Participants took the fluid intelligence test after completing the study phase (Lists 1–5). Then, after completing the fluid intelligence test, they completed the transfer task, creating a delay between the study and test phases of the experiment. (2) The pacing of the fluid intelligence test was fixed at 15 min to examine keep the delay between study and test constant for all participants.

- H7: In line with our results from Experiment 1a, we expected both value cues and specific schema instructions to support accuracy in the transfer task. We again expected a significant interaction between value and schema support such that value cues would provide a performance advantage when general instructions were given more so than when specific instructions were provided. We expected all conditions receiving some type of support to perform better than our control condition.
- H8: Like in other VDR experiments (Castel et al., 2002; Knowlton & Castel, 2022; Middlebrooks & Castel, 2018) and Experiment 1a, we expected to observe an effect of value on recall when value cues were present during encoding demonstrating value-directed remembering.
- H9: In line with our results in Experiment 1a, we expected higher JOLs to contribute to maintenance of recall performance across lists, whereas lower JOLs would be related to recalling fewer words with task experience.

### Method

#### Participants

Participants were 120 undergraduate students (age: 18–30 years, *M* = 20.08, *SD* = 1.61; gender identity: 91 women, 23 men, one nonbinary, one transgender, four prefer not to say) recruited from the UCLA Human Subjects Pool who were tested online and received course credit for their participation.^2^ On average, participants began learning English at 1.89 years (*SD* = 3.02). A sensitivity analysis based on the observed sample was conducted using G*Power (Faul et al., 2009). For a MLR with five predictors, assuming alpha = .05, power = .80, the smallest effect the design could reliably detect is η2 = .10.

#### Materials and Procedure

The design in Experiment 1b was identical to Experiment 1a. The materials and procedure in Experiment 1b were like those in Experiment 1a. However, all participants completed the RPM task after list 5 and before the final transfer task. On the RPM task, which served as the distractor task, instead of having unlimited time for completion, participants had a time limit of 15 min to complete the RPM task.

### Results

#### Recall

**Fig. 4:**
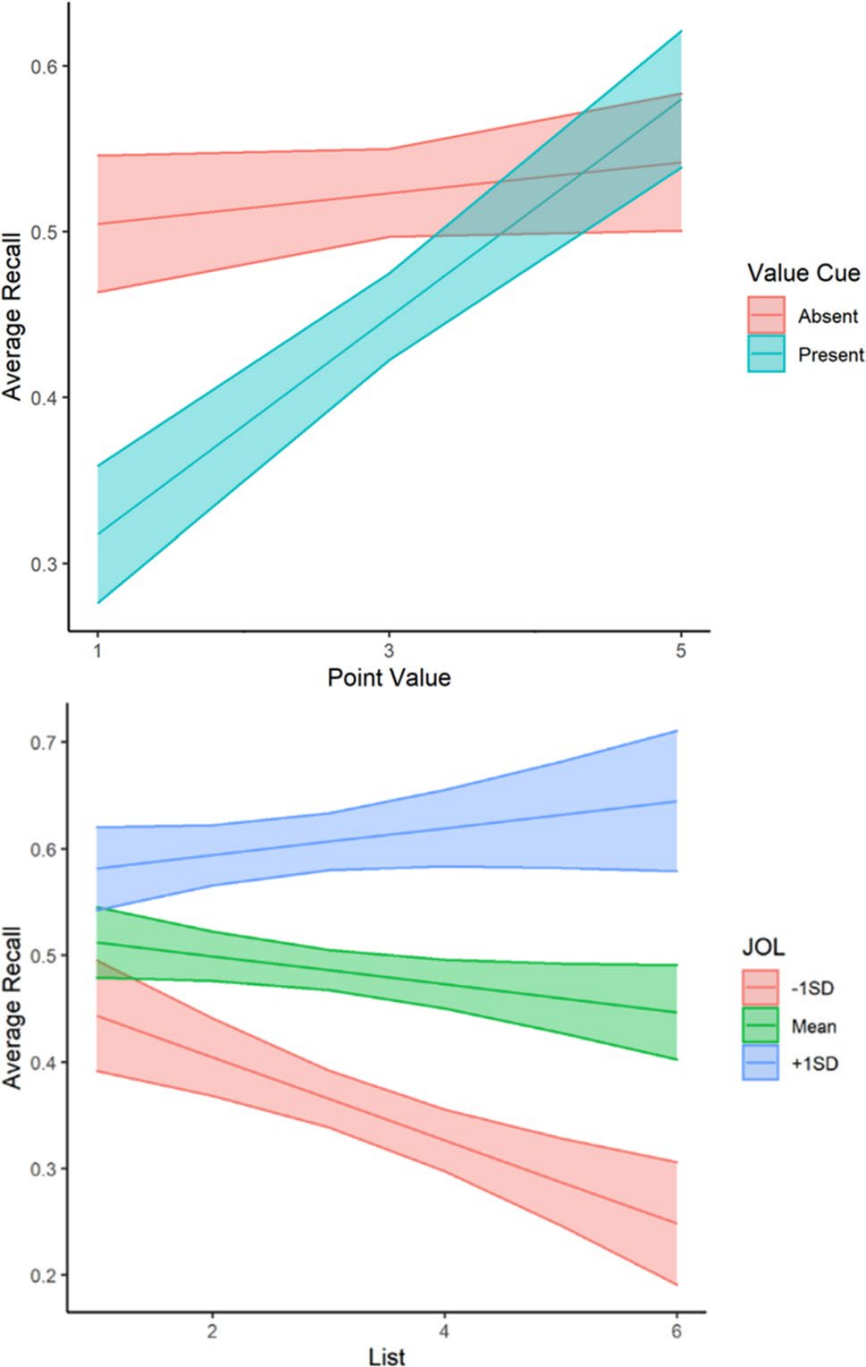
Recall performance in Experiment 1b. **Top graph:** Average recall as a function of list and average global judgment of learning. **Bottom graph:** Average recall as a function of point value and value cue condition. Confidence bands represent 95% confidence intervals for the predicted values of the mean

First, we sought to examine recall performance as a function of value cues, schema instructions, global JOL, list, and point value. We fit a MLR to model recall scores with value cues, schema instructions, point value, list, and global JOLs. We also included interaction terms to examine both how value cues impact the relationship between point value and recall and how metacognition impacts performance across lists. The model's explanatory power (*R*^*2*^) was .14. The model's intercept was at .49, *t*(1792) = 8.08, *p* < .001. The effect of point value, *b* = .01, *t*(1792) = 1.16, *p* = .25, was non-significant, suggesting that on average recall performance did not depend on the point value associated with each word. All other predictors were significant: The effect of specific schema instructions (coded as 1) was significant and negative, *b* = -.09, *t*(1792) = -4.81, *p* < .001, the effect of value cues (coded as 1) was significant and negative, *b* = -.24, *t*(1792) = -6.24, *p* < .001, and the interaction between value cues and point value was significant and positive, *b* = .06, *t*(1792) = 4.93, *p* < .001. Therefore, those receiving specific schema instructions recalled significantly fewer words on average compared to those receiving general instructions. Similarly, those receiving value cues during encoding recalled significantly less words on average, but they recalled significantly more high-value words compared to low-value words, *b* = 0.07, *t*(1792) = 8.13, *p* < .001, while recall did not depend on point value for those studying the words alone, *b* = 0.01, *t*(1792) = 1.16, *p* = .25 (see Fig. 4). Furthermore, the effect of average JOL was significant and positive, *b* = 0.24, *t*(1792) = 1.98, *p* = .048, the effect of list was significant and negative, *b* = -.07, *t*(1792) = -4.27, *p* < .001, and the interaction between JOL and list was significant and positive, *b* = .14, *t*(1792) = 3.95, *p* < .001. These findings suggest that holding all other predictors constant, on average, a one-unit increase in average JOL on the studied lists predicted a .24 unit increase in recall performance, and recall performance decreased by .07 with each additional list. However a simple slopes analysis revealed that the effect of list on recall was dependent on JOL such that those with average JOLs at the mean (*M* = .36, *SD* = .15), *b* = -.01, *t*(1792) = -1.93, *p* = .05 and 1 standard deviation below the mean, *b* = -.04, *t*(1792) = -3.98, *p* < .001, recalled fewer words with each additional list, while those with average JOLs 1 standard deviation above the mean recalled a similar number of words across lists, *b* = .01, *t*(1792) = 1.39, *p* = .16 (see Fig. 4).

#### Transfer of Learning

**Fig. 5:**
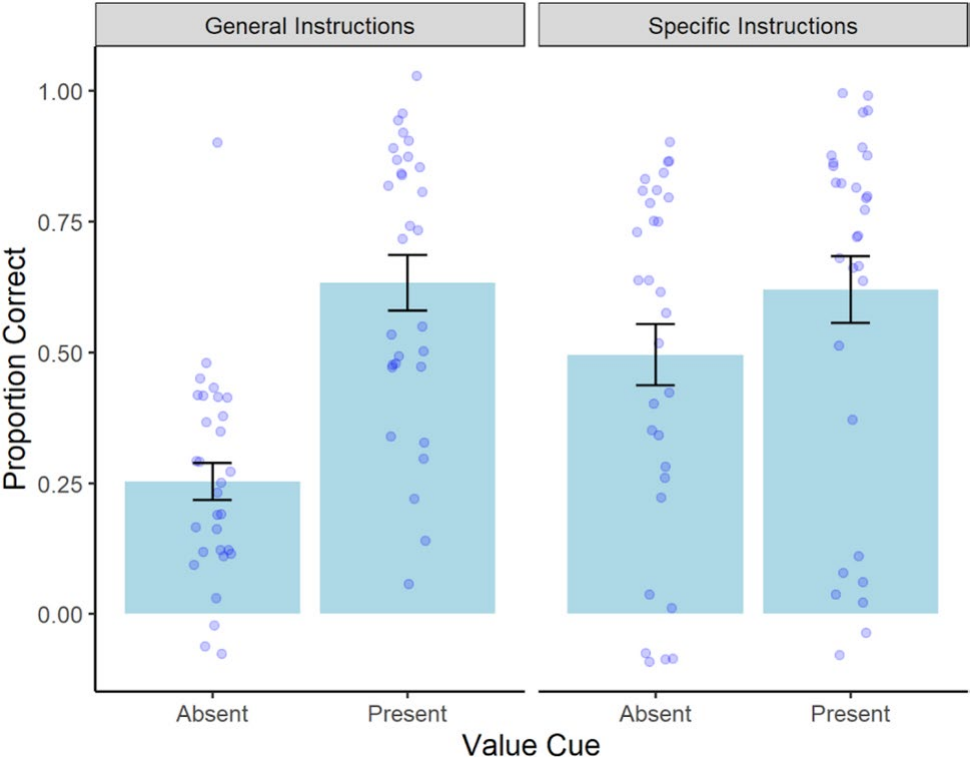
Average transfer scores as a function of value cue and schema instructions in Experiment 1b. Error bars represent the standard error of the mean

Next, we sought to examine transfer performance as a function of value cues, schema instructions, and point value. We fit a MLR to predict transfer of learning scores with value cues, schema instructions, and point value. We added an interaction term between schema instructions and value cue to test whether the effect of value cues on transfer performance was dependent on the type of schema instructions participants received prior to beginning the task. We also added an interaction term between value cues and point value to test whether the effect of value cues on transfer performance was dependent on whether value cues were present during encoding. The model's explanatory power (*R*^*2*^) was .17. The model's intercept was at 0.28, *t*(354) = 4.77, *p* < .001. The effect of point value, *b* = -.01, *t*(354) = -.64, *p* = .52, and the interaction between value cues and point value, *b* = .003, *t*(354) = .11, *p* = .91, were not significant. Therefore, transfer performance was not significantly influenced by how many points each word was worth upon recall, and the effect of point value did not depend on the presence of value cues during encoding. All other predictors were significant: value cues, *b* = .37, *t*(354) = 4.43, *p* < .001, schema instructions, *b* = .24, *t*(354) = 4.73, *p* < .001, and the interaction between value cues and schema instructions, *b* = -.26, *t*(354) = -3.53, *p* < .001. On average, participants receiving value cues during encoding performed significantly better on the transfer task than those who studied the words alone. Similarly, participants receiving specific schema instructions had significantly higher transfer scores than those receiving general instructions. Furthermore, the effect of value cues on transfer performance depended on the type of schema instructions that were provided at the beginning of the experiment. A simple slopes analysis revealed that when the schema instructions were specific, there was an effect of value cues on transfer performance, *b* = .12, *t*(354) = 2.43, *p* = .02; however, this effect was larger than when schema instructions were general, *b* = .38, *t*(354) = 7.41, *p* < .001 (see Fig. 5).

As in Experiment 1a, there were three possible point values participants were instructed to use as predictions of items on the transfer task, performing at chance on this task would be .33, or five items correctly paired with the appropriate point values. We conducted within-condition one-sample t-tests to examine whether each group performed better than chance and found that all groups receiving some form of support performed significantly better than chance on this task: Value Support (*M* = .63, *SD* = .33), *t*(89) = 8.69, *p* < .001, *d* = .92, Schema Support (*M* = .50, *SD* = .38), *t*(89) = 4.04, *p* < .001, *d* = .43, and Dual Support (*M* = .62, *SD* = .38), *t*(89) = 7.17, *p* < .001, *d* = .76. However, the No Support group who studied the words alone with general instructions performed below chance (*M* = .25, *SD* = .27), *t*(89) = -2.77, *p* = .007, *d* = -.29.

### Discussion

In Experiment 1b, we sought to replicate the findings from Experiment 1a and demonstrate that both schema instructions and value cues enhance learning in our novel VDL task even after a short delay between the study and the test. Consistent with Experiment 1a, results revealed that having specific schema instructions at the beginning of the task predicted higher transfer on the final test compared to receiving only general instructions (H7). We also replicated the finding that studying the words paired with values predicted higher rates of transfer on the final test (H7). Therefore, even after a short delay, participants were able to successfully demonstrate learning from the schematic reward structure when provided with either value or schema support (or both). In other words, the ability to learn the schematic reward structure and apply it in a novel test is preserved even when a short delay is introduced between learning and applying the new knowledge.

We also found that receiving specific schema instructions resulted in lower recall performance than receiving general instructions and that studying the words paired with value cues also was associated with lower recall performance. However, consistent with Experiment 1a, participants receiving value cues during encoding recalled significantly more high-value words, demonstrating strategic encoding and recall of words that would maximize their gains (H8). Furthermore, having higher JOLs was associated with higher recall performance suggesting that metacognitive monitoring plays a role in recall performance (H9).

## Experiment 2

In Experiment 1, participants receiving value cues and/or specific schema instructions were able to learn the schematic reward structure within the five word lists and applied their knowledge in the final transfer task. Given that one can learn the values of the studied categories through the schematic reward structure and apply them in a novel task, it is unknown whether one can speed up the learning process. In Experiment 2, to investigate whether participants could learn the schematic reward structure with fewer study trials and generalize the results of Experiments 1a and 1b to other categories beyond types of animals, we exposed participants to a new theme with each list. Specifically, participants studied six lists with each list having a unique theme (e.g., plants) with three categories (e.g., flowers, trees, herbs) and completed a transfer task after each list allowing us to investigate whether participants could adapt to a new theme with each list and learn its schematic reward structure.

In Experiment 2, participants did not receive specific schema instructions as we were interested in how they might learn the schematic reward structure with task experience with visible value cues compared to a control condition where no value support is provided. Prior research has shown that multiple tests can enhance learning, a phenomenon known as “the testing effect” (e.g., Karpicke & Aue, 2015; Storm et al., 2010). VDR research has shown that, with task experience, people learn to be more strategic and selective in their memory (Knowlton & Castel, 2022). Therefore, we expected participants to show an increase in transfer scores with task experience with the aid of visible value cues during encoding (H10). Because there was only one transfer trial in Experiment 1, we could not examine how well participants could perform once they were aware of the type of test to be expected.

Additionally, we further explored the relationship between metacognition and the transfer of learning. In Experiment 1 we found that making higher global JOLs during encoding the maintenance of recall performance with task experience. However, we did not have item-level measures of metacognition for either the encoding or transfer task. In Experiment 2, Participants provided item-level JOLs during the encoding phase and item-level confidence judgments after each transfer trial. We expected higher metacognitive judgments to be related to higher recall and transfer scores (H11). Finally, we expected participants studying words paired with value cues to recall more high-value words compared to the control group (H12).

### Method

#### Participants

Participants were 66 undergraduate students (age: 18–31 years, *M* = 20.64, *SD* = 2.43; gender identity: 60 women, six men) recruited from the UCLA Human Subjects Pool who were tested online and received course credit for their participation.^3^ On average, participants began learning English at 1.85 years (*SD* = 2.74). A sensitivity analysis based on the observed sample was conducted using G*Power (Faul et al., 2009). For a MLR with six predictors, assuming alpha = .05, power = .80, the smallest effect the design could reliably detect is η2 = .19.

#### Materials

Stimuli used in Experiment 2 consisted of 180 English nouns submitted to the ELP (Balota et al., 2007) database to generate measures of length (M = 5.80 letters per word, *SD* = 1.66), frequency in the HAL corpus (Lund & Burgess, 1996, M = 7.32 occurrences per million, *SD* = 1.60), and concreteness (M = 4.74, *SD* = 0.26). Participants were exposed to 12 lists and six themes, with one list of each theme used for the encoding phase and one used for the transfer task. The six themes used were animal names (categories: mammals, birds, and fish), food items (categories: fruit, vegetables, and meat), fashion items (categories: clothing, shoes, and jewelry), household items (categories: bedroom items, bathroom items, and kitchen items), vehicles (categories: air, land, and water), and plants (categories: flowers, trees, and herbs). See Appendix 2 for a complete list of materials.

#### Procedure

A 2 (Value: No Value Cue, Value Cue) × 6 (Category Theme: Animal names, Food items, Fashion items, Household items, Vehicles, Plants) design was used, with value being manipulated between participants and category theme manipulated within participants. Participants were informed that there were low-value (1 point), medium-value (3 points), and high-value (5 points) words and that their goal was to maximize their scores, the sum of values associated with the words they recall. During encoding, some participants viewed only the words (No Support) while others viewed the words paired with values of either 1, 3, or 5 (Value Support). These values were assigned based on category membership and value-category pairings were counterbalanced between participants. There were five items from each category on each list. Participants in the No Support condition were informed that they would not be able to see the values on the screen with the words. Additionally, participants made local JOLs after viewing each word, indicating how likely they would recall that item on a later recall test from 0 (not at all likely) to 100 (very likely). Immediately following the encoding phase for each list, participants completed the same distractor task used in Experiment 1 where they reordered number sequences. Following the distractor task, participants had 1 min to complete a free recall test by typing as many words as they could remember from the previously studied list and were given feedback in a form of their score out of a possible 45 points.

**Fig. 6:**
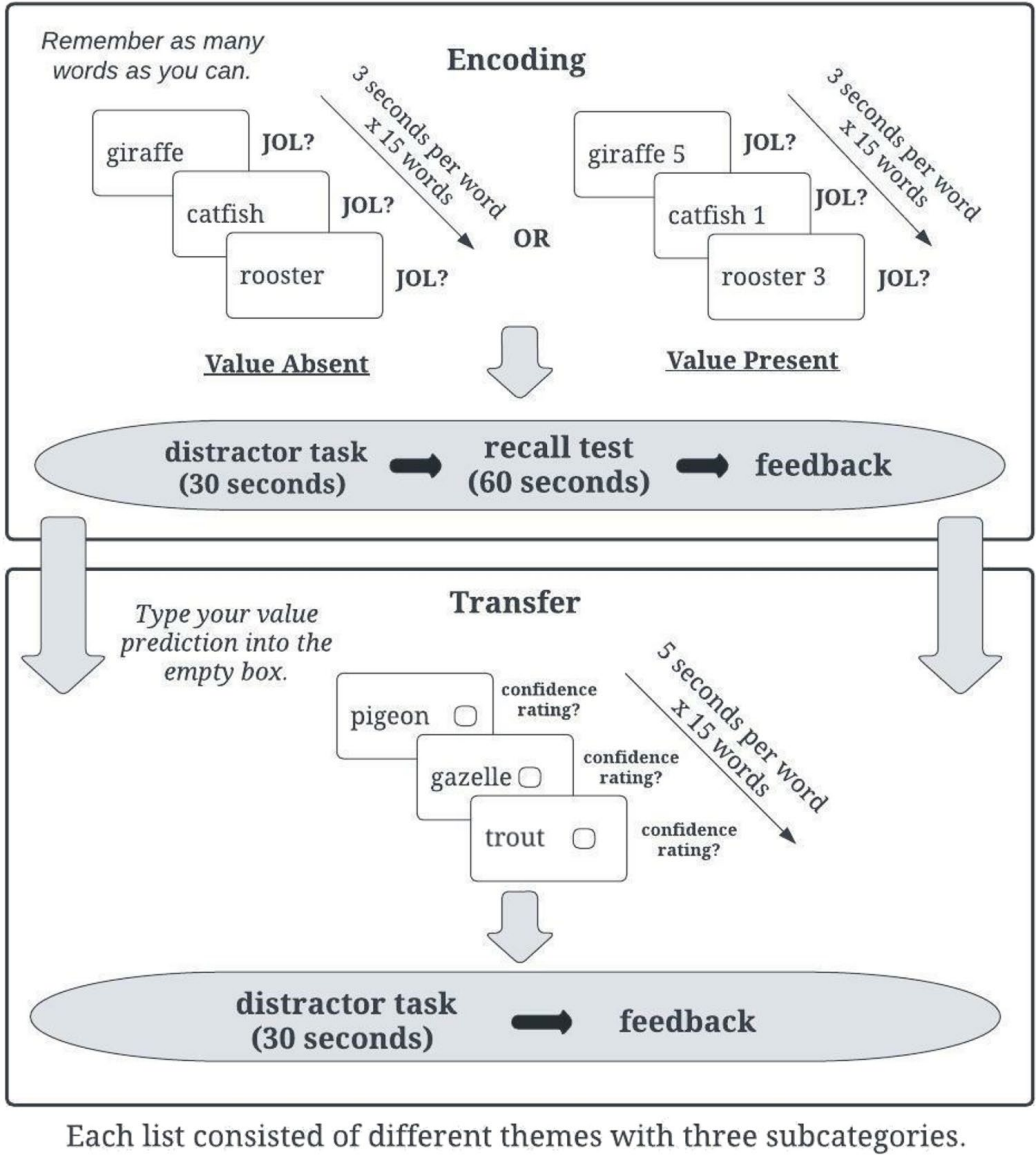
Procedure for the value-directed learning task in Experiment 2

Participants then proceeded to complete the transfer task for that list and were exposed to a set of new words belonging to the previous list’s categories. Each word appeared next to an empty box and participants were prompted to predict which value belonged with each word to measure their transfer of learning. Participants had 5 s to enter their value predictions into the box for each item. After predicting a value for each item, they were asked to rate how confident they were in their answers from 0 (not at all confident) to 100 (very confident). Participants followed this procedure for a total of six encoding-transfer phases. The complete procedure for Experiment 2 is illustrated in Fig. 6.

### Results

Measures used in the following analyses include local JOLs and confidence judgments, recall performance, and transfer of learning scores. All measurements were averaged across lists by associated point value before being entered into the analyses.

#### Recall

**Fig. 7:**
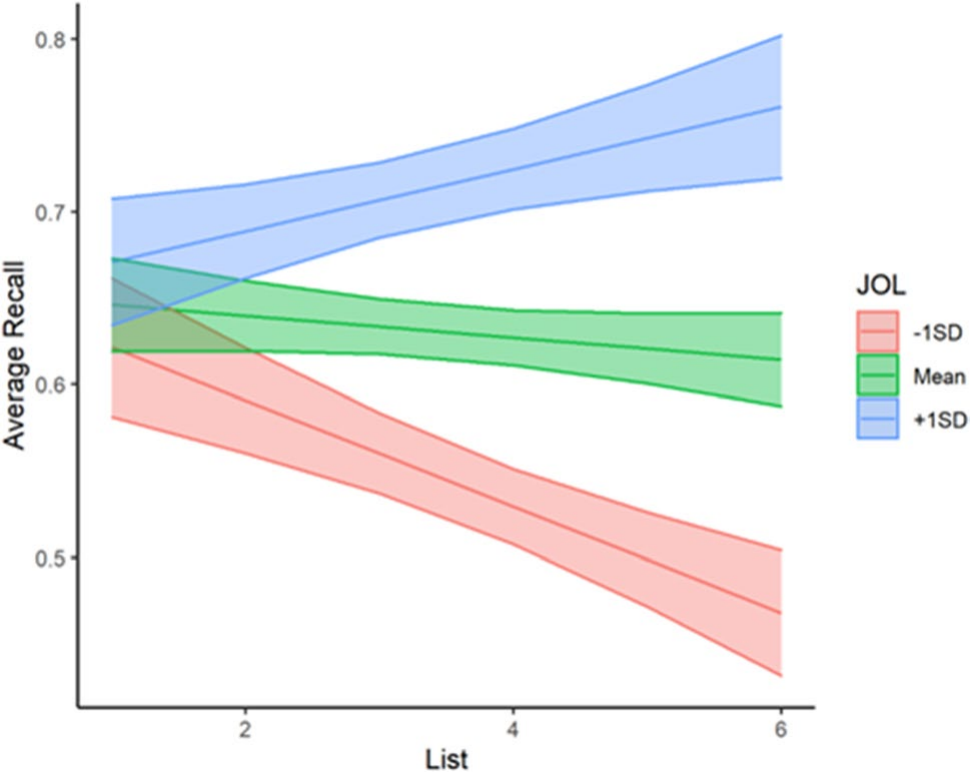
Recall performance in Experiment 2 as a function of average local judgment of learning and list. Confidence bands represent 95% confidence intervals for the predicted values of the mean

First, we sought to examine recall performance as a function of value cues, local JOLs, list, and point value. We fit a MLR to model average recall scores with value cues condition, point value, list, and local JOLs. We also included interaction terms to examine both how value cues impact the relationship between point value and recall and how local JOLs impact performance across lists. The model's explanatory power (*R*^*2*^) was .15. The model's intercept was at .59, *t*(1177) = 13.37, *p* < .001. The effects of value cue condition, *b* = .06, *t*(1177) = 1.82, *p* = .07, point value, *b* = -0.002, *t*(1177) = -0.30, *p* = .76, and JOL, *b* = .02, *t*(1177) = .23, *p* = .82, were non-significant, suggesting that receiving value cues at encoding did not significantly enhance recall. On average, recall performance did not change with the point value associated with each item. Furthermore, local JOLs did not significantly influence average recall. Additionally, the effect of point value on recall did not depend on whether value cues were present during encoding, *b* = .02, *t*(1177) = 1.91, *p* = .06. Because we had expected an effect of value on recall for the value support condition due to prior work in VDR and our results from Experiment 1, and this interaction was of theoretical interest, we probed the interaction by conducting a post-hoc simple slopes analysis which revealed that there was a significant effect of value on recall for the Value Support condition, *b* = .02, *t*(1177) = 2.39, *p* = .02, but not for the control condition, *b* = -.002, *t*(1177) = -.30, *p* = .76 (See Table 2 for descriptive statistics). However, recall did decrease with each additional list, *b* = -.05, *t*(1177) = -5.22, *p* < .001, and the effect of list on recall was influenced by local JOLs, *b* = .09, *t*(1177) = 5.13, *p* < .001. A simple slopes analysis revealed that those with JOLs at the sample mean (*M* = .48, *SD* = .26) recalled a similar number of words across lists, *b* = -.01, *t*(1177) = -1.39, *p* = .17. In contrast, those with JOLs 1 SD above the mean showed better recall performance with each additional list, *b* = .02, *t*(1177) = 2.63, *p* = .01 and those with JOLs 1 SD below the mean showed worse recall performance with each additional list, *b* = -.03, *t*(1177) = -4.66, *p* < .001 (see Fig. 7).

**Table 2:** **Mea**ns presented as proportion correct (with standard deviation in parentheses) for recall performance as a function of point value and condition in Experiment 2

Condition	1-point items	3-point items	5-point item
No Support	.57 (.31)	.59 (.30)	.57 (.29)
Value Support	.63 (.28)	.67 (.26)	.72 (.26)

#### Transfer of learning

**Fig. 8:**
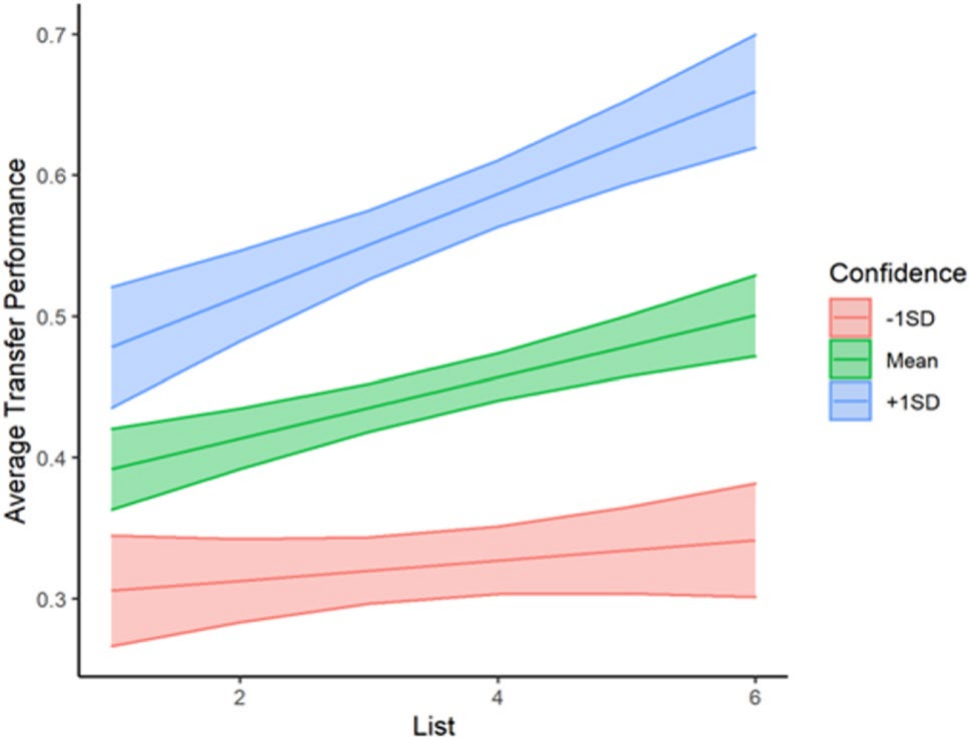
Transfer performance in Experiment 2 as a function of average confidence judgment and list. Confidence bands represent 95% confidence intervals for the predicted values of the mean

To examine average transfer performance as a function of condition (no value cue = 0, value cue = 1), local confidence judgment, list, and point value, we fit a MLR to model transfer scores with value cue condition, point value, list, and local confidence judgments. We also included interaction terms to examine both how local confidence judgments and value cue condition impacted transfer performance across lists. The model's explanatory power (*R*^*2*^) was .35. The model's intercept was at .10, *t*(1181) = 2.32, *p* = .02. The effects of list, *b* = -.01, *t*(1181) = -1.11, *p* = .27, and point value, *b* = .01, *t*(1181) = 1.90, *p* = .06, were non-significant, suggesting that on average, transfer performance did not increase with task experience and was not significantly impacted by the point value associated with each word. Furthermore, the effect of list did not depend on value cue condition, *b* = .02, *t*(1181) = 1.81, *p* = .07. As expected, studying the words paired with visible value cues resulted in significantly higher transfer performance, *b* = .24, *t*(1181) = 6.35, *p* < .001. Having higher average confidence judgments did significantly influence average transfer performance, *b* = .24, *t*(1181) = 3.73, *p* < .001 and the effect of list on transfer performance was dependent on average confidence judgments, *b* = .05, *t*(1181) = 3.01, *p* = .003. A simple slopes analysis revealed that those with confidence judgments 1 SD below the mean performed similarly across lists, *b* = .01, *t*(1181) = 1.08, *p* = .28. In contrast, those with confidence judgments at the mean (M = .50, *SD* = .30) showed better transfer performance with each additional list, *b* = .02, *t*(1181) = 4.50, *p* < .001, and as did those with JOLs 1 SD above the mean, *b* = .04, *t*(1181) = 5.16, *p* < .001 (see Fig. 8).

As in Experiment 1, chance performance on the transfer task would be 5 out of 15 correct (33%) as participants had three options for predicted values of each item. To test whether each group performed above chance, we conducted one-sample *t*-tests, which revealed that the group receiving value cues performed significantly better than chance, *t*(593) = 17.94, *p* < .001, *d* = .74, while the group studying the words alone performed significantly worse than chance, *t*(593) = -4.30, *p* < .001, *d* = -.18.

### Discussion

In Experiment 2, we expected transfer performance to increase with each list for participants in the value support condition as their prior knowledge of the semantic relationships between category items (McGillivray & Castel, 2017) paired with the value cues studied during encoding would enhance performance with task experience (Knowlton & Castel, 2022) (H10). As expected, participants were only able to learn the schematic reward structure when the words were paired with a visible value cue.

Additionally, the value category (i.e., low, medium, high) paired with each word did not significantly influence transfer of learning scores on average, suggesting that performance on the value-pairing task did not depend on the value category (H12). One difference between our design and typical VDR tasks is that we only used three values and these values each repeated five times on each list, while VDR tasks often use values ranging from 1 to 20 or 1 to 12 that do not repeat values within lists (Stefanidi et al., 2018). Therefore, participants in our new paradigm are learning a gist category associated with the word as opposed to an item-level value. Therefore, with this more discrete range of values compared to the continuous values in typical VDR tasks, we may have been underpowered to detect the value-directed remembering effect in this study. Even though we did not observe a significant interaction between value cue and condition in Experiment 2, we did probe the interaction post hoc using a simple slopes analysis and found that value did impact recall for the value support condition. We also found that average confidence judgments significantly impacted average transfer of learning scores such that having average to high confidence resulted in better performance with task experience while having lower confidence was related to no increase in performance with task experience (H11). Similarly, higher local JOLs were related to recalling more words with each list whereas having lower local JOLs were related to recalling fewer words with each list (H11).

## General discussion

The current study aimed to examine whether making participants aware of categories present within a series of word lists would facilitate a transfer of learning of the category-value pairings across lists. Prior work has shown that numerical values paired with words (Castel et al., 2002; Hennessee et al., 2019), item-location pairs (Siegel & Castel, 2018), and even faces (DeLozier & Rhodes, 2015) can enhance memory for important information. In Value-Directed Remembering (VDR), values paired with words facilitate the strategic control of memory, while in Value-Directed Learning (VDL) the value cues direct the learner’s attention to how the words on each list are related to one another. As we used well-known semantic categories, participants could learn the schematic reward structure of the lists when provided with either schema or value support, but not when provided with no support at all. As we discuss the results of the present experiments, we compare some findings across experiments when appropriate. As a supplement to these comparisons see Appendix 3, which contains summaries of the regression coefficients for predictors in our models by experiment and outcome variable.

The results of Experiment 1 suggest that both being aware of the schematic reward structure before encoding and receiving value support during encoding contributed to higher transfer of learning (H1 and H2) both with and without a short delay before encoding and transfer. However, in Experiment 1a, the effect of value cues on transfer was only beneficial when schema instructions were general (H3). On the other hand, when tested after a short delay in Experiment 1b, the effect of value cues on transfer was beneficial for both types of schema instructions, but this effect was stronger for general instructions (H7). In our transfer task, participants must make two decisions within the 5-second limit to properly predict each item’s value. First, they must categorize the word within the theme of the list, and then they must recall the value belonging to each item’s category. This process may utilize some form of working memory capacity (WMC), and relevant research has shown that performance in VDR tasks may be influenced by WMC (Griffin et al., 2019; Hayes et al., 2013; Knowlton & Castel, 2022) though other studies have reported little to no relationship (Castel et al., 2009;Cohen et al., 2014 ; Knowlton & Castel, 2022). It has been shown that people with high WMC demonstrate superior recall (Unsworth, 2007; Unsworth, 2016). Such individual differences in recall performance between people varying in WMC could be partially explained by the notion that individuals with low WMC are searching through a larger set of items than individuals with high WMC. Other work has looked at strategy use as a potential candidate for understanding the relationship between recall and WM and results revealed that people with high WMC reported using more effective strategies, such as grouping or sentence generation, than people with low WMC (Unsworth, 2016). Decision-making in our transfer task may require a heavier cognitive load than selectivity in a free recall test and having a measure of WMC could help determine the additional benefit of value when also receiving specific schema instructions after engaging in an unrelated task as this distraction may lead to some forgetting of the category-value pairings, especially when relying on knowledge of the schematic structure alone without value cues.

We also examined recall performance and found that studying words paired with values led to lower recall overall, but higher rates of high-value words recalled demonstrating selectivity (H4 and H8). Other work in VDR has shown that point values may cue the learner to engage in differential encoding strategies (Cohen et al., 2014; Knowlton & Castel, 2022). Additionally, having higher global JOLs resulted in stable recall across lists while having lower global JOLs resulted in a decrease in recall with task experience (H5 and H9). These findings suggest that value cues provide support in determining what is important to remember and participants are metacognitively aware of their performance. The act of selectively recalling words may be a mechanism through which participants can notice how the words are related, which could support performance on the transfer task. This strategy may become more conscious and explicit with task experience, consistent with current models of human reward pursuit (Bijleveld et al., 2012), and suggests a metacognitive mechanism that may help guide learning.

In Experiment 2, we tested participants after each studied list and the theme of the list changed after each study-test phase. We found that receiving value support resulted in significantly higher transfer of learning scores compared to studying the words alone (H10). Therefore, not only were participants in the Value Support condition able to learn and apply the schematic reward structures with only one study trial (compared to five trials in Experiments 1a and 1b), but they were also able to learn schematic reward structures of multiple lists each with a different theme, categories, and items.

Students are exposed to copious amounts of information and must be selective about what to study to be successful on assessments. Often, students struggle to decide what is most important to remember, though schemas and prior knowledge may guide what people tend to remember (McGillivray & Castel, 2017; Murphy & Castel, 2020, 2021). In Experiment 2, participants receiving value cues were able to adapt to new themes with each list and use both their prior semantic knowledge of the words and their task experience with the transfer test to learn not only which items were most important, but also what made an item important (i.e., category membership). Furthermore, as in Experiment 1, having higher Judgements of Learning facilitated recall during the encoding phase, suggesting metacognitive awareness of performance (H11). Additionally, because participants made confidence judgments after each item on the transfer tests, we also observed that confidence was positively related to transfer performance (H11). To achieve long-term learning in a domain, one must not only remember important facts and details but must understand how important concepts and themes are connected resulting in transferable knowledge (Bransford & Schwartz, 1999; Fries et al., 2020; Greeno et al., 1993; Renkl et al., 1996). Though this deep learning occurs over a long period, we can see in our experiments that assigning items point values based on categorical features facilitated predictions of novel items’ importance and metacognitive monitoring and control played an important role in this process.

A limitation of our stimuli is that some items were more prototypical of the categories they belong to than others (e.g., mammals: “giraffe” vs. “whale”). Natural prototypical stimuli are typically learned more quickly than their non-prototypical counterparts (Rosch, 1973). Additionally, we did not collect data on how familiar participants were with each item, which could be a factor in categorizing the words. Thus, we assumed prior knowledge of the words used in the study based on the demographics of our sample of fluent English speakers. Furthermore, while we conducted Experiment 2 in part to see whether participants could learn the schematic reward structure for categories other than types of animals, our results may not be generalizable beyond the specific well-known types of categories we chose for our experiments.

Numerous studies have found age-related differences in memory capacity; however, work using the VDR paradigm has demonstrated that older adults can be just as selective as younger adults and more selective than adolescents and children (Castel et al., 2011). However, our novel transfer task involves the binding of values to specific categories present on the word lists. The associative deficit hypothesis posits that older adults struggle with processing associative information (Naveh-Benjamin, 2000), thus future work should explore whether there are age-related differences in VDL tasks. Furthermore, Castel and colleagues (2011) demonstrated that children with Attention-Deficit/Hyperactivity Disorder (ADHD) Combined type display deficits in the strategic and efficient encoding and recall of important information in a VDR task. Attention to the value-category pairings in VDL may be important in facilitating performance on the word-value pairing transfer task. Future research could examine how individual differences may contribute to performance on the value-directed learning task, as well as how performance on this task may relate to performance on more standard value-directed remembering tasks in younger and older adults, to determine if similar attentional mechanisms and reward-based value-directed remembering mechanisms may contribute to performance.

Value-directed learning extends the VDR paradigm to category learning and demonstrates that the effect of value on recall persists even when there are more discrete value categories as opposed to continuous sets of values arbitrarily paired with words. We also explored how scaffolding instructions about to-be-studied items impacts the effectiveness of using value cues to identify a schematic reward structure across word lists. Using point values to group items may help learners identify what is most important to pay attention to and facilitate learning. Future work should explore how schematic reward structures could be applied to more realistic stimuli to aid in learning novel information.

## Summary

In the present study, we found that participants demonstrated the ability to learn the schematic reward structures of word lists designed around well-known categories relying on their prior knowledge of the relationships between the words and on value cues provided during encoding. We suspect that strategic control of memory motivated by value cues may direct the learner’s attention to the similarities between words associated with the same values. We have extended value-directed remembering mechanisms to a context in which strategic control of memory may lead to transfer of learning across lists. This work shows that using values to guide attention, promote strategic control of memory, and facilitate transfer of learning of the schematic reward structure with task experience may be an effective strategy to promote learning.

## Appendix 1: Items used in the word lists in Experiments 1a and 1b

**Table:**

Birds	Fish	Mammals
bluejay	anchovy	aardvark
dove	angelfish	alpaca
eagle	barracuda	baboon
emu	bass	badger
falcon	carp	buffalo
ferret	catfish	camel
flamingo	clownfish	dingo
goose	cod	donkey
gull	dory	elephant
hawk	eel	elk
kookaburra	flounder	gazelle
loon	guppy	giraffe
macaw	halibut	gopher
mockingbird	koi	hedgehog
owl	marlin	human
panot	minnow	hyena
partridge	perch	jaguar
peacock	roughy	koala
pelican	salmon	lion
penguin	sardine	mongoose
pigeon	seahorse	muskrat
quail	shark	otter
raven	snapper	puma
rooster	sole	raccoon
sparrow	stingray	reindeer
stork	sturgeon	squirrel
swallow	sunfish	turkey
swan	swordfish	whale
vulture	trout	wolf
woodpecker	tuna	zebra

## Appendix 2: Items used in the word lists in Experiment 2

**Table:**

Birds	Fish	Mammals
Parrot flamingo penguin emu hawk owl dove eagle raven vulture	shark tuna carp eel flounder halibut cod trout perch bass	aardvark baboon whale camel donkey badger muskrat mongoose lion koala

**Table:**

Fruit	Vegetables	Meat
apples oranges lemons grapes bananas pineapples plums cherries apricots peaches	corn broccoli celery peas spinach beets kale cabbage onions carrots	salami chicken turkey pork ham veal pepperoni beef quail bologna

**Table:**

Clothing	Shoes	Jewelry
pants dress coat skirt shorts jacket shirt jumpsuit jumper jeans	flip-flops sneakers boots loafers socks sandals slippers moccasins espadrilles heels	earrings necklace ring bracelet anklet choker brooch pins cufflink armlet

**Table:**

Bedroom	Bathroom	Kitchen
bed drawers wardrobe armchair sheets quilt hanger nightstand pillow mattress	toilet shower bathtub toothbrush shampoo razor urinal floss loofah mouthwash	fork strainer tupperware plate saucepan kettle cooker toaster fridge mixer

**Table:**

Air	Land	Water
spaceship rocket plane hovercraft jet balloon blimp helicopter drone glider	truck van wagon bus excavator pickup tractor taxi sedan motorcycle	jetski cruise lifeboat canoe boat kayak ferry sailboat yacht submarine

**Table:**

Herbs	Trees	Flowers
cilantro parsley dill chive rosemary sage thyme oregano basil mint	palm cedar birch sequoia redwood pine oak maple elm bamboo	tulip orchid peony violet rose poppy lily iris lilac daisy

## Appendix 3: Comparing findings across experiments

Regression coefficients in models predicting recall by experiment.

**Table:**

Predictor	Experiment 1a	Experiment 1b	Experiment 2
Specific Instructions	.002	-.09***	NA
Value Cue	-.12***	-.24***	.06
Point Value	.01**	.01	-.002
JOL	.34***	.24*	.02
List	-.03***	-.07***	-.05***
Point Value x Value Cue	.04***	.06***	.02
JOL x List	.05**	.14***	.09***

* *p* < .05, ** *p* < .01, *** *p* < .001

Regression coefficients in models predicting transfer by experiment.

**Table:**

Predictor	Experiment 1a	Experiment 1b	Experiment 2
Specific Instructions	.43***	.24***	NA
Value Cue	.27***	.37***	.24***
Point Value	-.01	-.01	.01
Fluid Intelligence	.002	NA	NA
List	NA	NA	-.01
Confidence Judgements	NA	NA	.24***
Point Value x Value Cue	.004	.003	NA
Specific Instructions x Value Cue	-.25***	-.26***	NA
Confidence Judgements x List	NA	NA	.05**
Value Cue x List	NA	NA	.02

* *p* < .05, ** *p* < .01, *** *p* < .001

## Footnotes

1 Exclusion criteria in all studies included removing participants from the final sample who admitted to cheating on a post-task questionnaire. No participants were excluded in Experiment 1a.

2 No participants were excluded in Experiment 1b.

3 One participant was excluded from analyses for admitting to cheating on a post-task questionnaire.

## Declarations

### Data availability

Data and survey materials are available via OSF (https://osf.io/6fxsq/).

### Code availability

No custom code or software was written for this project. Code to run analyses in R are provided in the OSF link.
